# Metabolism of Stem and Progenitor Cells: Proper Methods to Answer Specific Questions

**DOI:** 10.3389/fnmol.2019.00151

**Published:** 2019-06-13

**Authors:** Giuseppe Martano, Elena Monica Borroni, Egesta Lopci, Maria Grazia Cattaneo, Milena Mattioli, Angela Bachi, Ilaria Decimo, Francesco Bifari

**Affiliations:** ^1^IFOM—FIRC Institute of Molecular Oncology, Milan, Italy; ^2^Humanitas Clinical and Research Center, Rozzano, Italy; ^3^Department of Medical Biotechnology and Translational Medicine, University of Milan, Milan, Italy; ^4^Nuclear Medicine Unit, Humanitas Clinical and Research Hospital—IRCCS, Rozzano, Italy; ^5^Laboratory of Cell Metabolism and Regenerative Medicine, Department of Medical Biotechnology and Translational Medicine, University of Milan, Milan, Italy; ^6^Laboratory of Pharmacology, Department of Diagnostics and Public Health, University of Verona, Verona, Italy

**Keywords:** stem cell, cell metabolism, neural stem cell (NSC), indiced Pluripotent Stem Cells (iPSCs), metabolic flux analysis (MFA), metabolomics (OMICS), cell reprogramming, pharmacology

## Abstract

Stem cells can stay quiescent for a long period of time or proliferate and differentiate into multiple lineages. The activity of stage-specific metabolic programs allows stem cells to best adapt their functions in different microenvironments. Specific cellular phenotypes can be, therefore, defined by precise metabolic signatures. Notably, not only cellular metabolism describes a defined cellular phenotype, but experimental evidence now clearly indicate that also rewiring cells towards a particular cellular metabolism can drive their cellular phenotype and function accordingly. Cellular metabolism can be studied by both targeted and untargeted approaches. Targeted analyses focus on a subset of identified metabolites and on their metabolic fluxes. In addition, the overall assessment of the oxygen consumption rate (OCR) gives a measure of the overall cellular oxidative metabolism and mitochondrial function. Untargeted approach provides a large-scale identification and quantification of the whole metabolome with the aim to describe a metabolic fingerprinting. In this review article, we overview the methodologies currently available for the study of *in* *vitro* stem cell metabolism, including metabolic fluxes, fingerprint analyses, and single-cell metabolomics. Moreover, we summarize available approaches for the study of *in vivo* stem cell metabolism. For all of the described methods, we highlight their specificities and limitations. In addition, we discuss practical concerns about the most threatening steps, including metabolic quenching, sample preparation and extraction. A better knowledge of the precise metabolic signature defining specific cell population is instrumental to the design of novel therapeutic strategies able to drive undifferentiated stem cells towards a selective and valuable cellular phenotype.

## Introduction

The intertwining relationship between cell energy metabolism and function has been reported since the early 1960s (Cohn and Hirsch, 1960; Oren et al., 1963). Increasing evidences suggest that cells adopt distinct metabolic signatures in different functional conditions, thus leading to cell metabolism as an emergent research field (Pearce and Pearce, 2013) and creating novel exciting opportunities for future therapeutic applications (O’Neill et al., 2016).

Cell metabolism describes the biochemical phenotype of the cell. However, it is not a fixed cell property since it undergoes continuous and highly dynamic modifications to adapt cells to changes in energy requirement, fuel availability, biomass production, reductive-oxidative status, post-translational and epigenetic settings. Moreover, cell metabolism connects the extracellular microenvironment (i.e., availability of nutrients, such as glucose and oxygen) with the functional cellular need (for example, cell growth, migration, or synapse formation; Formaggio et al., 2010; Dibble and Manning, 2013; Ochocki and Simon, 2013; Yuan et al., 2013).

Players of cell metabolism consist of substrates, enzymes, cofactors, reaction products and by-products, all involved in biochemical reactions. The quantity, velocity and compartmentalization of these reactions specify cellular functions. Importantly, cell metabolism should not be considered independently of the genetic and proteomic cellular context. Indeed, all these features converge to define the final cell phenotype and function (Metallo and Vander Heiden, 2013). A very simple way of describing the cellular status at a given time would be to consider it as the result of a one-way chain of events: gene expression determines protein content that in turn defines the metabolic status of the cell. However, it has been well characterized that all of these events are closely and intricately regulated, and proteins control both DNA transcription and metabolic reactions. More recently, the capability of metabolites to regulate gene expression, as well as protein activity and distribution, has been described (Lu and Thompson, 2012; Sperber et al., 2015). Consequently, to fully define and potentially modulate cellular activities, a multilayer picture of the cellular phenotype should include the characterization of cell metabolism, in addition to the study of gene expression and protein function.

Not surprisingly, cells in different conditions, such as quiescent stem cells (SCs), amplifying precursors and differentiated cells, show dramatic changes in their cellular metabolism (O’Neill et al., 2016). However, unlike other cells in the tissues, SC population are usually rare and intermingled with other cell types of the niche. Moreover, in the stem cell niche quiescent SC, expanding precursors and differentiated cells coexist together, and the proportion of these SC phenotypes may vary depending on the tissue condition (e.g., health or disease). Therefore, there are specific constraints imposed by working with stem cells. Due to their rare distribution, metabolic analysis obtained from the whole tissues may not be always representative of the SC metabotypes. On the other hand, classic available methods to sort specific SC population cannot entirely be applied since both fluorescence activated (FACS) and immunomagnetic cell sorting requires too long time for sample preparation and processing prior to metabolic quenching, the time in which cells undergo mechanical stress and quickly change their metabolism. The recent development of single-cell metabolomics, multimodal *in vivo* imaging and novel biosensors, that allows real-time metabolism at single cell level in living samples, may offer new opportunities to specifically describe stem cell metabolism. Hence, appropriate methods need to be applied for the study of SC metabolism. In this review article, we will provide an up-to-date overview of the different techniques for the investigation of cellular metabolism of SCs, highlighting the peculiarities, strengths and limitations of each methodology.

Understanding cell metabolism of SCs and of their differentiated progenies provides unique insights for the identification of molecular hubs capable of integrating the multiplicity of signaling underlying these processes, and driving stem cell quiescence, expansion and differentiation. Rewiring cell metabolism is nowadays an attractive and innovative strategy for developing novel and effective drugs able to restore stem cell function, and eventually, help to heal the pathological phenotype.

## Cell Metabolism of Undifferentiated and Differentiated SCs

During embryogenesis, SCs symmetrically expand their number, blood perfusion is still incomplete, and proliferating cells relay mostly on glycolysis for their metabolic needs (Ito and Suda, 2014; Gu et al., 2016). Subsequently, a proportion of cells undergo differentiation, and this process often implies an increase in their metabolic needs (Prigione et al., 2015). SC differentiation generally requires morphological and functional changes. As an example, during development, neural stem cells (NSCs) self-renew, expand the number of committed progenitors, migrate to the cortex, and differentiate into mature neurons that functionally integrate within the tissue (Bifari et al., 2017a; Pino et al., 2017; Kempermann, 2019). NSCs persist in selected regions of the adult mammalian brain (Bifari et al., 2009, 2015; Decimo et al., 2011; Bond et al., 2015). NSCs have multipotent differentiation potentials and differentiated cells greatly modify their cellular morphology (Decimo et al., 2012a,b). Differentiating oligodendrocytes progressively expand cellular branching, reaching a mean of about 20 branching/cell (Butt et al., 1994; Dolci et al., 2017). All these differentiation stages are accompanied by specific changes in cellular metabolism (Lange et al., 2016; Knobloch and Jessberger, 2017; Beyer et al., 2018). Neuronal differentiation, synaptic transmission, generation and conduction of action potentials are highly metabolic-demanding cellular activities (Laughlin et al., 1998). Accordingly, differentiated neuronal cells need to adapt their metabolism towards a more efficient oxidative metabolism (Lange et al., 2016; Beckervordersandforth et al., 2017). Indeed, the adult brain accounts for more than 20% of the body oxygen consumption.

Increasing evidence demonstrate that plasticity in energy metabolism is a crucial regulator in shaping the balance between self-renewal potential and lineage specification (Folmes et al., 2012; Ito and Suda, 2014; Prigione et al., 2015). In particular, a proper quality control of mitochondrial function has been recently highlighted as a key factor in SC maintenance and commitment (Shyh-Chang et al., 2013). In order to demonstrate hematopoietic SC (HSC) repopulating capacity, HSCs are kept in a quiescent state, where they exhibited higher glycolysis rate and lower mitochondrial respiration than committed progenitor cells (Chandel et al., 2016; Roy et al., 2018). The disruption of this metabolic checkpoint leads to the loss of quiescence and to a reduced regenerative capacity, and directs HSCs towards lineage commitment where the displacement to mitochondrial metabolism (mitochondrial oxidative phosphorylation) is essential, in order to rapidly respond to the increased demand of energy (Vannini et al., 2016). Importantly, the mammalian Target Of Rapamycin (mTOR), one of the most important regulators of mitochondrial function *via* the increase in mitochondrial biogenesis, is required for the active cycling of HSCs losing stemness (Chen et al., 2008). Mitochondria also act as the leading site for the production of Reactive Oxygen Species (ROS), and ROS accumulation finally contributes to the defective functioning of HSCs and their loss of stemness. Accordingly, ROS clearance exhibits a positive effect on HSC recovery of stemness (Chandel et al., 2016; Roy et al., 2018). In this scenario, autophagy, or rather mitophagy, a self-degradative process involved in the energy balance (Mizushima and Komatsu, 2011), plays an essential role in the reversion of metabolically active HSCs to quiescence by clearing healthy but active mitochondria, thus preventing the induction of (epi) genetic programs that lead to HSC commitment, and thereby supporting the maintenance of healthy hemeatopoiesis (Riffelmacher and Simon, 2017; Jin et al., 2018).

Alterations of the redox state are also crucial in the regulation of proliferation and differentiation of neural progenitors. Mitochondrial dynamics regulate stem cell fate by modifying ROS signaling, which in turn increases the nuclear factor erythroid 2–related factor 2 (NRF2) gene expression promoting neuronal differentiation (Beckervordersandforth et al., 2017). Furthermore, fatty acid oxidation (FAO) is specifically upregulated in quiescent neural SCs (Folmes et al., 2012), and it has also been involved in the control of neural SC proliferation (Shyh-Chang et al., 2013).

Although the maintenance of SCs and the generation of differentiated lineages are undoubtedly conditioned by cellular metabolism, to date, less is known about the regulation of the differentiation potential of SCs by exogenous nutrient availability (glucose, glutamine and fatty acids, but also lipids and nucleotides) and by their intracellular pathways. The glucose transporter 1 (Glut1) and the glutamine transporter ASCT2 are highly upregulated during HSC erythroid differentiation. As a matter of fact, downregulation of ASCT2 or the block of glutamine metabolism abrogate HSC erythroid differentiation, diverting towards a myeloid fate (Montel-Hagen et al., 2008; Pouzolles et al., 2016).

Notably, metabolic fuels do not solely function as sources of ATP (Folmes and Terzic, 2016; Oburoglu et al., 2016) as well as cellular metabolism not merely describes how the cell gets its energy. The specific metabolic signature of the cell accounts for its unique phenotype and functions. Fuel sources actively impact on cell signaling and transcription (Yuan et al., 2013; Saxton et al., 2016; Bifari and Nisoli, 2017; Bifari et al., 2017b) by integrating metabolic and epigenetic signals (Ochocki and Simon, 2013). For example, the glycolytic metabolite phosphoenolpyruvate stimulates the transcriptional activity of the Nuclear Factor of Activated T cells (NFAT) that regulates Mitofusin 2, a key player in the mitochondrial fusion, trafficking and turnover, modulating the maintenance of HSCs with lymphoid, but not myeloid, lineage potential (Ho et al., 2015; Luchsinger et al., 2016). Similarly, reprogramming of neuronal metabolism has been shown to greatly protect the brain from stroke (Quaegebeur et al., 2016).

Somatic cell reprogramming to pluripotent state is also associated with a drastic rewiring of cellular metabolism. The metabolic shift to glycolysis is an obligatory step for somatic cell reprogramming (Folmes et al., 2011), and inhibition of glycolysis prevents the reprogramming of the induced pluripotent stem cells (iPSCs). Interestingly, early after reprogramming, an oxidative phosphorylation burst is also required. Therefore, to successfully go to the pluripotent state, iPSCs synergistically enhance both glycolysis and oxidative phosphorylation. Several factors appear to be associated with this reprogramming-induced hybrid energy metabolism. Following reprogramming, the up-regulation of Zic3 and Esrrb genes activate glycolytic metabolism independently of hypoxia-inducible factors, while Esrrb, but not Zic3, mediates oxidative phosphorylation activation (Sone et al., 2017). Up-regulation of estrogen-related receptors is also involved in the oxidative phosphorylation burst in both human and mouse iPSCs (Kida et al., 2015).

In conclusion, increasing evidence demonstrate that SCs in different functional states adopt distinct metabolic properties and signatures, and that imposing specific cellular metabolism can drive SC self-renewal and differentiation (Quaegebeur et al., 2016). A deeper understanding of the molecular mechanisms underlying metabolic control of the SC properties is therefore of great biological interest. More importantly, improved knowledge on cell metabolism will be critical for the development of novel exciting therapeutic strategies targeting SC self-renewal, proliferation and differentiation for the regenerative medicine (Decimo et al., 2012a; Shyh-Chang et al., 2013; Ito et al., 2019).

## Methods to Study SC Metabolism: A User’s Guide

### Different Experimental Approaches for Different Questions

Understanding the intracellular metabolism of SCs is a pivotal task to clarify mechanisms that govern their maintenance, proliferation, migration and differentiation in response to biological stimuli. Resolving heterogeneity between different cellular phenotypes is still a challenging task that requires high sensitive methodologies. In this contest, Nuclear Magnetic Resonance (NMR)- and mass spectrometry (MS)-based methodologies are the preferred choices due to their ability to generate high-throughput data from a limited volume of sample with relatively low concentrated metabolites.

**Figure 1:**
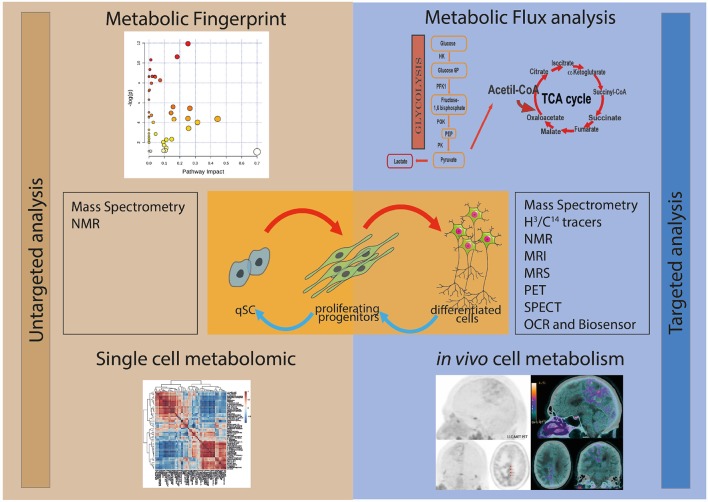
Schematic representation of the different experimental approaches for the study of the metabolism of stem and progenitor cells. In the left panel are depicted the untargeted metabolic analysis, metabolic fingerprint (upper left) and single-cell metabolomics (lower left). The study of the whole metabolite content (untargeted metabolic analysis) of the samples can be performed by using mass spectrometry (MS) and nuclear magnetic resonance (NMR). In the right panel are figured the targeted metabolic approaches, metabolic flux analysis (upper right) and *in vivo* cell metabolism (lower right), which focus on the study of metabolites possibly involved in a specific pathway. Several techniques can be applied to study targeted metabolic analysis, including MS, NMR, magnetic resonance imaging (MRI), magnetic resonance spectrometry (MRS), positron emission tomography (PET), single photon emission tomography (SPECT), oxygen consumption rate (OCR) and Biosensor.

Two main approaches can be used for studying stem cell metabolism: the untargeted and the targeted metabolic analyses (Figure 1). Untargeted metabolic assessment implies the study of all the metabolites that can be measured, thus giving an overall description of the metabolic state of samples. This metabolic fingerprint provides an accurate description of the metabolite abundance present at a given time inside the cell. Targeted metabolic analysis focuses on the study of a subset of metabolites possibly involved in a specific pathway. In addition, the overall assessment of the intracellular oxygen consumption rate (OCR) gives valuable information on the oxidative metabolism and mitochondrial function.

The investigation of the metabolic dynamics, i.e., metabolic flux analysis, is a complementary approach to the quantification of metabolites and it is often required to fulfill the missing information that cannot be provided by metabolic fingerprint. In fact, the profile of metabolites is very powerful in describing differences in pathways and nodes among samples, but it does not explain the biological mechanism(s) responsible for these differences. As an example, the metabolic profile can show a drastic difference in the concentration of a metabolite between two different experimental conditions. However, without the study of the dynamic fluxes, it is not possible to predict whether the change is due to a modulation in the biosynthesis, to degradation of the metabolite, to a different mobilization from segregated storage or to the activation of alternative pathways.

Available techniques effectively analyze the cellular metabolism of homotypic cell population *in vitro* or of tissues *ex vivo*. Although encouraging attempts have been made, the assessment of the cellular metabolism at single cell level remains not entirely resolved. Furthermore, the study of the *in vivo* cellular metabolism remains challenging. The use of detectable probes in living tissue allows the evaluation of the cellular metabolic activity. Although the specificity and sensitivity of the *in vivo* techniques are remarkably high, the spatial resolution (0.12 mm; Blüml and Panigrahy, 2013; Shah et al., 2013; Stucht et al., 2015) is too large for resolving the metabolism of single cells.

### Nuclear Magnetic Resonance (NMR) vs. Mass Spectrometry

Two major technologies are used for metabolomics analysis, i.e., NMR and MS. The two platforms have different limits and advantages deeply discussed elsewhere in the literature (Ellis et al., 2007; Pan and Raftery, 2007). The critical issue regarding SCs is the availability of biomaterial for analysis and the related sample volumes and concentrations. MS analysis is an obliged choice for the aforementioned limits, due to its higher sensitivity compared with NMR, but it does not imply that NMR analysis should be avoided. In fact, the two methods are very comparable and complementary with a highly different coverage. There are limited examples of concomitant use of both techniques in metabolomics, but the scientific community is recognizing more and more the need to complement the technical limits by coupling these two technologies online. Although this association will probably be the method of choice in the foreseeable future, the tools for this type of analysis, e.g., software and hardware handling both instruments online, and integrated data analysis, are still in their infancy or lacking. The most popular methodology in metabolomics is based on liquid chromatography coupled with MS. This is due to advances in material science and instrumental precision and sensitivity. In liquid chromatography, a major advancement was achieved by the introduction of core-shell particle technologies (Fekete et al., 2012). Compared with classical stationary phases, core-shell particles have lower mass-transfer dispersion, thus resulting in a lower height of theoretical plates and enhanced separation efficiency. As a result, this type of stationary phase allows for narrowing of the peak width, and as a consequence, increases the peak intensities (Martano et al., 2014). These characteristics are well exploited thanks to the development of chromatographic platforms that can handle high pressure (>1,000 bars) and are coupled to high resolution mass spectrometers that are able to sustain a scan rate in the millisecond ranges. Of particular interest, the ability to scale down the instrumental platform to nanoUHPLC coupled with nanoESI-MS gives the possibility to analyze the metabolome using a limited number of cells *per* sample (commonly between 100 and 500 cells), with high metabolic coverage. This methodology allows for further downscaling of sample investigation to single cell analysis, but this choice is not costless in term of metabolic coverage, separation and sample handling, as discussed later.

### Metabolic Fingerprint

Questions:

Is the stem cell population A different from the stem cell population B? Is the stem cell population changing its phenotype from condition X to Y? What are the main nodes and the most relevant pathways of the cellular metabolism of a specific stem cell population?

Metabolomics is a broad and sensitive method to detect differences in cellular metabolic states. Notably, this metabolic assessment is related to a very precise time and space, and it can be considered as a snapshot of the global metabolome of the sample analyzed. Metabolic fingerprint may provide valuable information in understanding the impact of different experimental conditions on the overall metabolic state of cells or tissues. Metabolomics provides precise information on the intra- or extra-cellular metabolite levels. However, metabolic fingerprint does not give any indication on the cause of the measured variations of metabolites. This is very important to consider when planning the experiment. If we need to study how cells or tissue modify their overall metabolome, or more precisely the metabolite abundance of specimens, untargeted metabolomics is the gold standard technique. Furthermore, based on the observable changes in absolute or relative intracellular metabolite levels, we can define the metabolic pathways and nodes that are modulated in different specimens. What we cannot infer, however, is the reason why the measured alterations occur. Increase in substrate (i.e., fructose 6-phosphate) and decrease in a product (fructose 1,6-bisphosphate) may be due to an altered enzymatic activity (phosphofructokinase-1), as well as to an altered consumption of the product (increase/decrease glycolysis) or excessive production of the substrate (hexokinase activity). To further understand the biological reasons from which the differences stem, complementary technique assessing the metabolic dynamics (metabolic flux analysis) should be used.

Several issues need to be taken into account when designing the experimental plan for metabolic investigation. Sorting the desired cell population is arguably the most critical step in this type of analysis. Especially for metabolites with very fast turnover rate and low in abundance, the time required for sample preparation negatively influences the biological readout. The speed of sampling becomes even more critical when planning the investigation of kinetic profile using labeled standards, which require a tight sampling time to avoid data dispersion.

#### Metabolic Quenching

**Figure 2:**
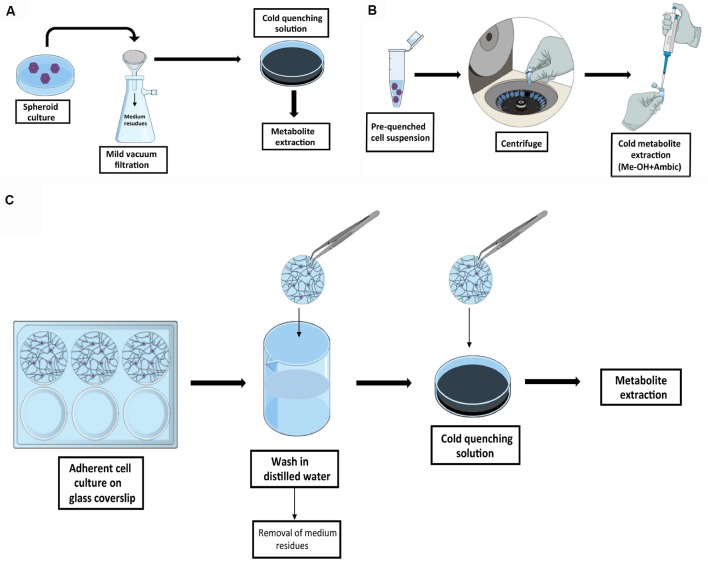
Graphic summary of recommended quenching workflows for SCs. Schematic representation of the quenching methods for cell in suspension by fast filtration adapted from Bennett et al. (2008) **(A)** and by cold quenching prior to sample collection as described by Sellick et al. (2009) **(B)**. In Panel **(C)** is outlined the quenching methods for adherent SCs by culturing cells on glass coverslips as described by Martano et al. (2015).

In *in vitro* experiments, the selection of the proper sampling method is linked to the type of culture condition selected, i.e., adherent or in suspension cultures (Figure 2). SCs can be grown as spheroids. Although there are no protocols specifically designed for the investigation of the metabolism of SCs in this condition, similar protocols originally developed for bacterial culture are easily adaptable to this purpose. Fast filtration, on filters followed by the transfer of the filter in cold organic quenching solution, as developed by Rabinowitz’s group, provides a robust pipeline and has been successfully used in this type of analysis (Bennett et al., 2008; Abu Dawud et al., 2012). In this approach, batch culture is slowly dripped onto a nylon filter membrane placed on the top of a vacuum flask, with a moderate-week vacuum applied in order to minimize mechanical stress. Additional washing step can be applied in this stage to further remove medium residues. Filters are then transferred in a separated well containing a quenching solution, and metabolites are extracted for analysis. The method is also suitable for profiling the kinetic flux using labeled substrates (Yuan et al., 2008). Fast filtration avoids the delay that would be introduced by typical harvesting procedures based on pelleting by centrifugation. A major concern against pelleting is due to the time required for the entire procedure prior to metabolic quenching, time in which the cells undergo mechanical stress. In that case, the extracted metabolites will likely represent the response of the cells to the harvesting procedure rather than the original biological phenotype. On the other hand, pelleting can be efficiently used if the metabolism is already quenched without damaging the cell integrity as in the method described by Sellick et al. (2009). The protocol was developed for the investigation of Chinese hamster ovary and mouse myeloma NS0 cell lines and utilized a quenching solution composed by 60% methanol in ammonium bicarbonate, added to avoid membrane disruption, and maintained at −40°C. This method does not require the use of filters and the procedure is very straightforward, but the major drawback is that it cannot be easily adapted for dynamic labeling profile analysis.

In adherent NSCs cultivated on coated surface, e.g., poly-L-lysine coating, similar issues need to be taken into account. In this case, cell harvesting should be avoided (e.g., trypsinization and pelleting) in favor of simultaneous quenching, and extraction in cold solvents. The limiting step when working with adherent cells is that a fast and complete removal of culture medium is required prior to quenching and extraction. A strategy to overcome this issue is presented in the protocol developed by the Vorholt group, in which cells were grown on glass coverslips (Martano et al., 2015), as it is conventionally done for experiments in microscopy. In this protocol, coverslips containing the cell culture are removed from the original medium, dipped and carefully tilted in a separated beaker under a steam of water, and immediately transferred in a cold quenching solution. This allows the fast removal of the cells of interest from the original medium and the subsequent fast wash in a steam of distilled water before quenching, with a minimal perturbation of the cell culture. The same procedure can be applied when performing dynamic labeling profile analysis by replacing the distilled water with a minimal medium without the metabolite of interest, thus avoiding the stochastic influence of the carry-over from initial medium.

*Ex vivo* experiments and metabolic profiling from tissues are probably the most challenging approach to work with. Strategies such as heat-stabilization and snap-freezing of the tissue of interest after dissection are usually avoided due to the effects of *post-mortem* degradations (Mulder et al., 2016). Instead, *in situ* freezing or funnel freezing are preferred, because they can be performed under anesthesia while the tissue maintains blood flow and oxygenation (Mulder et al., 2016), thus avoiding perturbation of the metabolic state. After the metabolism has been quenched, a selected micro-dissected region of interest can be sampled. The challenge is then to choose the proper analytical technique to analyze the selected region. The major concern relates to the under representation of SCs in the tissues. Therefore, data obtained from homogenized tissues, although the selected tissue may be enriched with SCs, will not be representative of the metabotype that those cells exhibit. Other approaches, based on mass spectrometry imaging (MSI) and single cell analysis may be applied instead, as discussed in later paragraphs.

#### Sample Preparation and Extraction Tips

Another critical step in performing metabolic profile analysis is the selection of the extraction solvent in which the metabolites are solubilized before analysis. Due to the heterogeneity of chemical-physical properties of the metabolome, which includes highly hydrophilic as well as hydrophobic compounds, it is difficult, if not impossible, to achieve the complete metabolome extraction using a single technique. Second, the extraction procedure must be compatible with the analytical methods selected for analysis and should avoid the artificial production of compounds that alters the original metabolite concentration. A classic example of generated artifacts is the ratio between ATP, ADP, AMP, analyzed by LC-MS in positive mode with the most common mobile phase i.e., water, acetonitrile with 0.1% formic acid. In this case, the acid hydrolysis will promote the dephosphorylation of the metabolites, which will be converted in the precursors (e.g., ATP will turn in ADP, and ADP in AMP), and therefore, the analysis will return an overestimation of less phosphorylated compound at the expenses of highly phosphorylated ones. Commonly, a mixture of methanol, acetonitrile and water can be used to both cold quench the metabolism and extract the metabolites of interest. The ratio between the solvents can be arranged based on the hydrophobicity/hydrophilicity of the metabolites of interest. The organic content has the advantage of promoting protein precipitation and sample extraction in one-step but causes instability in reverse-phase chromatography. This limit can be circumvented by solvent exchange where the samples are dried e.g., lyophilized, and samples are re-dissolved in the appropriate solvent. This also gives the possibility of storing the samples of interest for prolonged periods but has the disadvantage of introducing potential perturbation/degradation in oxygen-reactive metabolites. Therefore, while a generalized approach cannot be drawn, a wise choice of the available options must be taken in to account based on the species of interest. For example, for labile compounds such as Acetyl-CoA, the presence of acetonitrile and formic acid may compromise the compound recovery. In that case, after quenching, a mild solvent exchange using lyophilization or nitrogen steam followed by aqueous extraction can enhance extraction efficiency for Acetyl-CoA.

### Single-Cell Metabolomics

Questions:

What is the heterogeneity of stem cells? Are the experimental conditions affecting the cell phenotype in a different way and/or time?

Resolving the heterogeneity of cell populations is an attractive field in several omics discipline including metabolomics. The possibility to scale-down the analysis down to a single cell is of particular importance in the field of cancer research, where cancer SCs subpopulations are very likely responsible for the origin of metastatic phenotypes and contribute to chemotherapy resistance and relapse (Tu, 2010; Zenobi, 2013). Despite the recent innovations in single cell metabolic analysis, the major threat not entirely fixed yet is the time required for sampling. Metabolism is fast and dynamic and keeping cells in their natural environment as much as possible before sample processing remains the most critical aspect of analysis. Methods based on the cultivation of isolated cells are suffering from the artificial environment, which is projected into the metabolic profile. Few methods allowing single cell resolution in MS have been reported in the latest years. Initially, successful results were achieved exploiting very large cells, such as neurons in the peripheral nervous system, using capillary electrophoresis coupled with mass spectrometry (CE–MS; Nemes et al., 2013). This method was further expanded by coupling off-line patch clamp electrophysiology and CE-MS, and metabolic analysis (Aerts et al., 2014). However, as other methods were developed for this purpose, the size of the interested cell represents a limitation, and unfortunately, due to their small number, SCs often fall below the minimum analyzable size. In the latter case, limited suitable methods have been reported in the literature. One of these approaches has been developed by the Masujima’s group and is named Live Single Cell Mass Spectrometry (LiveSCMS; Mizuno et al., 2008). This method used nanotips, which can be handled to collect the cytosol of a selected cell under the microscope, the nanotips are then mounted as emitters for MS analysis using a nanoESI source. This method allows working with adherent cells with the size limit linked to the internal diameter of the selected nanotips i.e., 10 μm. Major drawbacks of this methodology are linked to the complete manual sampling of the specimen of interest. Another suitable method, recently published, combines different techniques, such as Fluidic-force Microscopy (FluidFM) and MALDI-MS, for analysis (Guillaume-Gentil et al., 2017). A major advantage compared to LiveSCMS resides in the ability to trace the sampling using atomic force microscopy, which gives real-time information on the position of the tip. After sampling, the cell content can be released on microarrays for mass spectrometry (MAMS) and analyzed with MALDI-MS. Both LiveSCMS and FluidFM-MS have the advantage of the ability to work at a single cell level, with high metabolic coverage without retrieving the target cells from their environment prior to sampling, but are limited by the time required for sampling. Complementary to those approaches, MSI can be used down to subcellular space with the possibility of analyzing high number of cells with a limited timeframe, but with a limit in the number of inspected metabolites (Kompauer et al., 2017).

#### Data Normalization

Another challenging aspect in the metabolomic analysis of SCs is the choice of data normalization and standardization, which is highly dependent on the selected methodology. In this regard, single cell untargeted metabolomics may be considered the most reliable approach. In fact, in that case, the number of cells used in the dataset is known, and the outcome from the metabolomics analysis can be used to standardize the sample, e.g., by dividing for the sum of intensity of all the detected metabolites in order to minimize technical variations. Untargeted analysis on bulk cells populations requires the introduction of constrains for data normalization. A common approach is to normalize the metabolome with the total protein content. This approach is particularly convenient with cold organic extraction solutions, in which proteins are insoluble and can be collected by centrifugation, thus efficiently separating the proteome in the pellet from the metabolome in the supernatant, while summed intensities of metabolites can still be used to minimize technical errors. However, not all the workflows allow the evaluation of protein content. Moreover, the total amount of protein per cell is largely influenced by the cell size, and therefore, not applicable for SCs analysis with different grades of differentiation. When none of these options are available, i.e., in targeted analysis where total protein content cannot be determined or utilized, another possibility is to relay on housekeeping metabolites (Mizuno et al., 2017). Their selection heavily depends on experimental conditions. Unfortunately, due to the high networking of metabolic pathways, it is impossible to predict whether a metabolite will be indirectly influenced from the experimental conditions. Therefore, the stability of the selected normalizer needs to be empirically evaluated. For example, we can consider an experimental condition in which the energetic state of SCs is perturbed. In this example, ATP, often used as normalizer, is unsuitable for this scope. However, it is worth noting that the vast majority of metabolites are transformed into each other through biochemical reactions and ATP is not an exception. In that case, if biosynthesis and degradation of nucleosides are unaffected by the experimental conditions, the sum of concentrations between ATP, ADP and AMP should remain constant and therefore can represent a robust normalizer (Martano et al., 2016).

### Metabolic Flux Analysis

Questions:

What are the specific metabolic pathway differences between two stem cell populations? Is a specific metabolic pathway modified by experimental condition in the stem cell population of interest? Which is the fate of a specific metabolic substrate of the sample?

Complementary to metabolic fingerprint analysis, the study of the dynamic between metabolites is becoming more and more required in order to fulfill the missing information on metabolic variations and rearrangements in the studied models. Although metabolic flux analysis does not provide information on the abundance of the metabolite present in the specimen, it can give an accurate indication on the direction and rate of metabolite conversion (flux) and whether these properties change between experimental conditions. Changes in the metabolic flux can be measured using radioactive tracers (De Bock et al., 2013). Following administration to the culture medium of 5-3H-Glucose or 3H-9, 10-palmitic acid, the quantification of H_3_-H_2_O produced is a measure of the glycolytic and the fatty acid oxidative fluxes. Similarly, the measure of 14C-CO_2_ produced by the cell culture following the addition in the medium of 6-14C-glucose, U-14C glutamine or 1-14C-glucose, is the quantification of glucose, glutamine and pentose phosphate oxidations. The quantification of all these metabolic paths allows the calculation of the contribution of each path to the overall ATP production. However, with radioactive tracers, it is not possible to follow the precise fate of the substrate. The dynamic labeling profile and fate of a substrate metabolism can be achieved by switching one of more nutrient factors with their heavily labeled counterparts and then follow over time the incorporation of the labeled substrate downstream in the metabolism e.g., replace glucose with ^13^C labeled glucose and follow the ^13^C entering in the glycolysis, pentose phosphate pathway or TCA cycle. Following ^13^C labeled substrates (tracers), the quantification of the ^13^C products is performed (^13^C tracer analysis). Notably, different fluxes can be studied following administration of a ^13^C labeled substrates by analyzing incorporation of ^13^C labeled metabolites at different time points. After seconds to minutes from the administration of ^13^C glucose, we will assess glycolytic intermediates and in the following hours, we will be able to quantify ^13^C labeled metabolites branching from more distant pathways such as lipid biosynthesis. The study of the metabolic flux of a specific pathway requires a *metabolic steady state*, implying constant intracellular metabolite levels and intracellular metabolic fluxes. Concerning ^13^C labeled substrate, enrichment into a given metabolite is stable over time relative (isotopic steady state).

Metabolic flux analysis can be used at different experimental stages including *in vitro*, *ex vivo* and *in vivo* analysis of metabolism. Three major considerations must be taken into account for planning at best this type of analysis, i.e., selection of the substrate, experimental time windows, and minimization of system perturbations. The first point strictly relates to the core pathways of interest, and therefore, choosing the substrates to be replaced with the labeled counterparts should reflect the nearest entry point of the core pathway of interest. For example, when studying gluconeogenesis, providing labeled pyruvate or lactate will directly feed the pathway of interest enabling more robust readouts compared with substrates, which are more distant in metabolic terms (Jang et al., 2018). The second point, the experimental time windows, is probably the most difficult to predict, but the most important for a correct readout. As a general statement, metabolic incorporation spans from millisecond to hours based on the dynamic activity of a given pathway *plus* the vicinity to the given substrate, and it is affected by various factors, such as diffusion into tissues in *ex vivo* experiments or tissue availability in *in vivo* studies. For a correct interpretation of the dynamic, we need to tune the time points in order to have the initial phase of incorporation as well as the late point of incorporation when the labeling reaches a plateau. Combined together, the initial slope with the total mobilized pool of each metabolite will give us the possibility to evaluate the turnover rate, and therefore, assess the kinetic of metabolic fluxes. The third point is linked to the strategy that can be followed to minimize the perturbations based on the type of experiments that are planned to be performed. In this case, it is important to avoid alteration of the nutrient factors during the experiment when possible. As an example, in *in vitro* experiments, it is important to work without varying the media composition between media with or without labeled nutrients. This approach should be preferred in favor of *de-novo* addition or supplementation of the labeled substrate because the latter approach will change the microenvironment in which the cells are growing, making impossible to distinguish whether the dynamics observed are linked to the microenvironment changes or not. It is also important to minimize carryover between different conditions. This possibility can be strictly avoided *in vitro* by choosing a proper sample preparation strategy as previously discussed, but not in *ex vivo* or *in vivo* experiments where this option should be taken in to account when interpreting the data or by comparing the substrate levels during the experiment e.g., *in situ* micro dialysis (Watanabe et al., 2018).

### *In vivo* Cell Metabolism

Questions:

How is the metabolism of a cell population or of a tissue *in vivo*? Is the metabolism of a selected cell population or of a tissue changed following different experimental paradigms or in diseases?

Cellular metabolism evaluation can be performed *in vivo* by means of multimodal imaging modalities. This implies the use of easily detectable probes tracking the metabolic activity of groups of cells in living tissue. Based on the characteristics of the probes or vehicles used, it is possible to apply MRI (magnetic resonance imaging), MRS (magnetic resonance spectroscopy) or PET (positron emission tomography) and SPECT (single photon emission tomography). The resolution of the abovementioned modalities decreases significantly while passing from conventional MRI (0.12–0.24 mm) and PET (1–3 mm) to SPECT (10 mm) and MRS (1–10 cm^3^; Blüml and Panigrahy, 2013; Shah et al., 2013; Stucht et al., 2015). Higher resolution levels can be instead achieved in animal studies by means of micro-PET, SPECT and/or MRI (Koba et al., 2013). Yet, none of these modalities is capable of imaging at the cellular level.

*In vivo* metabolic imaging with MRS allows the identification of protons (1H) or other specific isotopes present in normal compounds involved in the main metabolic pathways of living cells: this is the case for instance of 17O (oxygen-17), 23Na (sodium-23), 19F (fluoride-19), and 31P (phosphor-31; Herholz et al., 2007). One of the most well-known examples is represented by MRS in the identification of tumor cells thanks to the specific spectroscopic peaks of choline (Cho), N-acetyl aspartate (NAA), lactate and/or the Cho-to-creatinine (Cho/Cr) ratio that can be used as surrogate markers of tumor growth and malignancy (Lopci et al., 2015). Choline is a precursor of phosphatidylcholine, a component of the cell membrane, directly related to cell-membrane turnover. Typically, a choline peak reflects the presence of proliferative tissue consistent with a glioma or malignant tumor (Lopci et al., 2015). NAA is the second most abundant metabolite in the central nervous system and is related to the synthesis of neuropeptide N-acetylaspartylglutamate (NAAG), involved in the neurotransmitter release and neuronal communication (Moffett et al., 2006). Lactate is related to increased glucose uptake and lack of oxygen supply, hence it becomes detectable on MRS solely in tumor cells (Wang et al., 2017). Also, creatinine is a marker of cellular energy metabolism and its ratio to choline (Cho/Cr) tends to reflect the aggressiveness or grade of tumor tissue (Herholz et al., 2007; Toyooka et al., 2008). While using the same principle as mentioned above, MRS can also lead to the molecular stratification of tumors, such as gliomas, by searching very specific metabolites along the pathway. This can be the case for the oncometabolite 2-hydroxyglutarate, which serves as a surrogate marker of the isocitrate dehydrogenase (IDH1 and IDH2) mutation status (Choi et al., 2012). 1H-MRS has been also used to identify metabolites enriched in rodent neural stem cells and was used to indirectly quantify adult human neurogenesis (Manganas et al., 2007).

Furthermore, diffusion-weighted MRS (DWI-MRS) has been used for metabolic imaging. More precisely, it was introduced to combine the *in vivo* exploration of intracellular structures and physiology at the same time, allowing the detection and quantification of the diffusion properties of the metabolites involved (Valette, 2015). More recently, cell tracking with superparamagnetic iron oxide has been applied to study other cell types, including mesenchymal stem cells (Bengel, 2011). Iron particles can be detected at micromolar concentrations, thus permitting a very high resolution imaging of the cellular diffusion (Bulte, 2009).

**Figure 3:**
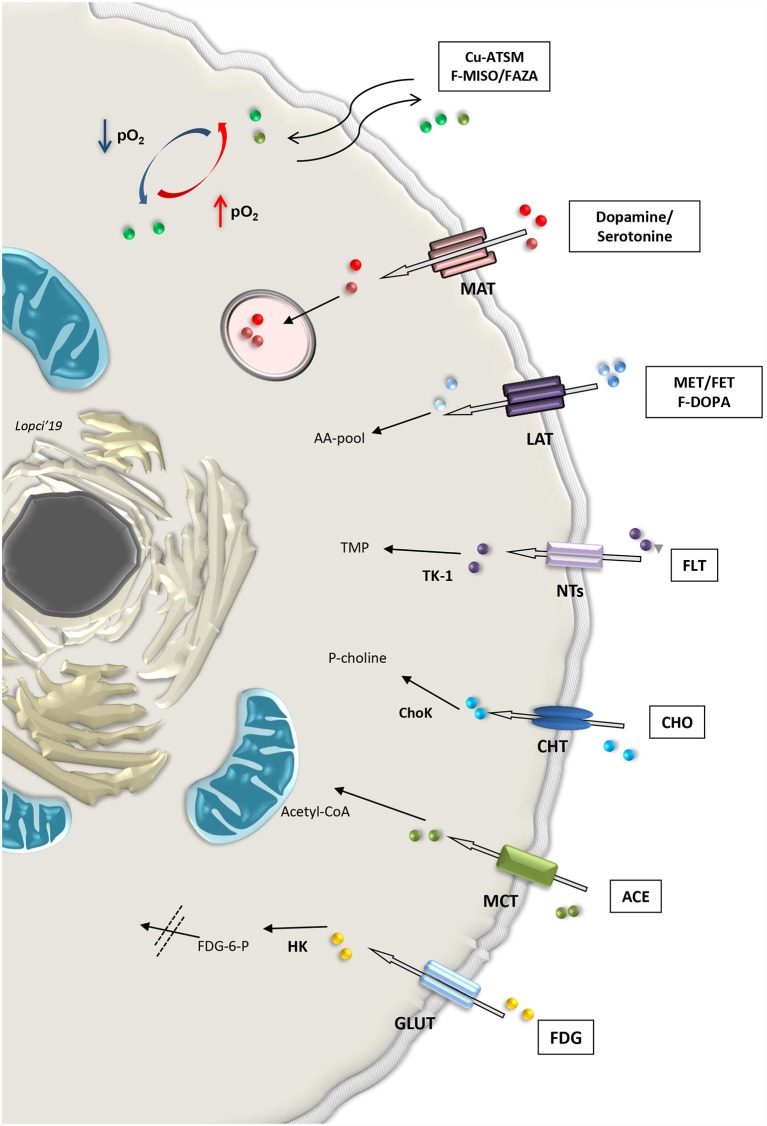
Summary of selected metabolic pathways detectable non-invasively with radiolabeled compounds applicable with PET imaging in clinical practice. Abbreviations: Cu-ATSM, Copper(II)-diacetyl-bis(N(4)-methylthiosemicarbazone; F-MISO, fluoromisonidazole; FAZA, Fluoroazomycin Arabinoside; MAT, monoamine transporter; MET, methionine; FET, fluoroethyltyrosine; LAT, large amino acid transporter; F-DOPA, fluoro-3,4-dihydroxyphenylalanine; AA-pool, amino acid pool; FLT, Fluorothymidine; NTs, nucleoside transporter families; TK1, thymidine kinase-1; TMP, thymidine monophosphate; CHO, choline; CHT, choline transporter; ChoK, choline kinase; P-choline, phosphocholine; ACE, acetate; MCT, monocarboxylate transporter; FDG, fluorodeoxyglucose; GLUT, glucose transporter; HK, hexokinase; FDG-6-P, fluorodeoxyglucose-6-phosphatase.

Cell tracking can also be performed by means of direct labeling with radioactive nuclides for imaging in SPECT or PET (Bengel, 2011). First, endothelial and hematopoietic progenitor cells labeled with 111In-oxine, 99mTc-exametazime or 99mTc-hexamethylpropyleneamine oxime (HMPAO), were used for scintigraphic detection on SPECT, along with 2-deoxy-2-(18F) fluoro-d-glucose (18F-FDG) for PET imaging (Aicher et al., 2003; Bengel, 2011). During the process, blood and blood components are taken from the patient and handled to isolate the required cell line that needs to be labeled. All these steps are extremely delicate and require caution to maintain the cells alive after labeling with the radioactive tracer (de Vries et al., 2010; Bengel, 2011). Imaging procedure will allow the functional assessment of those specific cells within the organs and tissue investigated. More broadly, metabolic radiopharmaceuticals are used on PET to visualize both normal and pathological mechanisms. Theoretically, all hallmarks of stem or differentiated cell population can be detected with imaging by simply radiolabeling the molecule or the active element (“probe”) in the focused pathway (Alam et al., 2015). Also, labeled antibodies can be used to identify the component characterizing the pathway. For instance, it is possible to image CD133 expression in SCs by means of the detection of a specific epitope called AC133, using the copper-64 labeled PET compound 64Cu-labeled AC133-monoclonal antibody (Gaedicke et al., 2014; Alam et al., 2015). As a non-invasive imaging modality, PET can be applied in the preclinical and clinical setting to measure site-specific accumulation of various metabolic “probes” or radiopharmaceuticals (Laing et al., 2010; Lopci et al., 2014, 2015; Figure 3).

The first and most widely used PET tracer is 18F-FDG, a glucose analog labeled with fluoride-18. Similarly to glucose, FDG is transported into the cells by means of the facilitated diffusion glucose transporters (GLUTs), and in particular GLUT1 (Navale and Paranjape, 2016). Once inside, the tracer is phosphorylated by the enzyme hexokinase (HK), which determines the entrapment of 18F-FDG within the cells (Laing et al., 2010). This process is significantly increased in both anaerobic glycolysis (“Pasteur effect”) and aerobic (“Warburg effect”; Lopci et al., 2014). To study tissue oxygenation, it is possible to use the PET tracers belonging to the fluorinated nitroimidazole derivatives or nitroimidazole family compounds, such as 18F-fluoromisonidazole (18F-FMISO), 18F-fluoroazomycin arabinoside (18F-FAZA), 18F-fluoroerythronitroimidazole (18F-FETNIM), 18F-fluoroetanidazole (18F-FETA) and 18F-2-nitroimidazol-pentafluoropropyl acetamide (18F-EF5; Lopci et al., 2014). Nitroimidazole family tracers are lipophilic and diffuse passively through cell membrane. Once inside, they are reduced into R-NO_2_ radicals by the nitroreductase enzyme (NTR). When the cell is not oxygenated (pO_2_ <10 mmHg), the reduction process continues to R-NHOH compounds that bind covalently to intracellular molecules. This determines the ultimate entrapment of the tracer in the cell (Lopci et al., 2014). More peculiar tracers for hypoxia imaging are represented by copper (60, 61, 62, 64 Cu) labeled diacetyl-bis (N4-methylthiosemicarbazone; Cu-ATSM), which follows another oxido-reductive process (Takahashi et al., 2000), and radiolabeled antibody anti-carbonic anhydrase IX (or CAIX), also known as antibody Grawitz250 (G250; Takahashi et al., 2000). This later radiopharmaceutical tracks CAIX, which is a transmembrane enzyme and one of the downstream targets of hypoxia-inducible factors 1α (HIF-1α). More specifically, its role is to hydrolyze the carbondioxide (CO_2_) into carbonic acid (H_2_CO_3_) and stabilize intracellular pH (Shuch et al., 2008).

Combined energetic and metabolic imaging can be offered by other tracers used in PET (Figure 3). Radiolabeled carbon-11 acetate (11C-acetate) is one example of multifunctional tracers, reflecting both oxidative metabolism and fatty acid synthesis pathways. The molecule is transported into the normal cell through the monocarboxylate transporter (MCT) and converted in acetyl-CoA by the acetyl-CoA synthase (ACeS) present in the cytosol and mitochondria (Klein et al., 2001; Croteau et al., 2016). In the cytosol, acetyl-CoA pathway leads to the synthesis of cholesterol and fatty acids, which are incorporated into cell membranes, while in the mitochondria, it leads to complete oxidation to 11C-CO_2_ and H_2_O (Lopci and Fanti, 2013). The higher the Krebs cycle activity, the higher 11C-acetate clearance. The tracer can also be used as a marker of fatty acid synthase, a key enzyme for oxidative metabolism and fatty acid synthesis pathway (Witney et al., 2014; Croteau et al., 2016).

Another key element for the evaluation of membrane phospholipids is represented by radiolabeled choline (18F- or 11C-choline), which is the precursor of phosphatidylcholine (Zeisel, 1981). The molecule is an essential component also for the synthesis of neurotransmitters, such as acetylcholine, or lipid mediators, such as platelet-activating factor (Challapalli and Aboagye, 2016). It is rapidly taken up into the cell by the choline transporter (CHT) and phosphorylates by CHK (Choline kinase) to phosphocholine (P-Choline). P-Choline is further phosphorylated by the enzyme cytidylyltransferase to cytidine diphosphate-choline (CDP-choline) and other metabolites until it is incorporated into the cell membrane (Gibellini and Smith, 2010). In highly proliferative cells, the uptake of radiolabeled choline reflects the levels of membrane turn-over, which is associated with increased choline transport and utilization, due to the increased CHK expression in undifferentiated and stem cells (Jansen et al., 2006; Glunde et al., 2011; Challapalli and Aboagye, 2016).

Cellular proliferation is visible directly by means of the fluorinated thymidine analog 18F-3′-deoxy-3′-18F-fluorothymidine (18F-FLT). This tracer enters the cell by means of the nucleoside transporters (NTs) and is then phosphorylated by the enzyme thymidine kinase 1 (TK1). This process determines the entrapment of the tracer within the cell and its uptake is a direct reflection of the TK1 activity and cellular S-phase, since FLT, unlike thymidine, is only marginally (<2%) incorporated into the DNA chain (Been et al., 2004). 18F-FLT uptake correlates with Ki-67 (Croteau et al., 2016).

Amino acid metabolism found several representatives in PET imaging. Radiolabeled methionine (11C-L-methyl-11C-methionine, 11C-MET) or tyrosine (18F-fluoroethyltyrosine, 18F-FET) are two of the most widely used compounds with this regard. These molecules are transported in the cell *via* the large amino transporter (LAT1) and once inside, their uptake reflects protein metabolism (Lopci et al., 2014, 2015). Metabolism of methionine and tyrosine greatly affect SC biology, including iPSC stemness and differentiation potential (Shiraki et al., 2014) and mesenchymal stem cells (Higuera et al., 2012). Tracer accumulation in the differentiated brain cells is almost negligible and significantly increases in proliferating cells, such as glioma-initiating cells (Law et al., 2019).

Other molecules, like 18F-dihydroxyphenylalanine (18F-DOPA), follow the same transportation within the cell as 11C-MET or 18F-FET, although 18F-DOPA is not simply an amino acid but also a precursor for catecholamine metabolism, and therefore, quite relevant for a subset of neuronal populations (Koopmans et al., 2009). Once inside the cell, 18F-DOPA is decarboxylated *via* the enzyme aromatic acid decarboxylase (AADC) to 18F-dopamine, which is probably then transported into secretory vesicles by vesicular monoamine transporter (VMAT; Luxen et al., 1992). Catecholamine and their intermediate products, such as dopamine or serotonine and their radiolabeled forms, enter directly into the secretory vesicles after being transported in the cell through the MCT system (Figure 3).

### Oxygen Consumption Rate (OCR) and Biosensors

Mitochondrial function greatly impacts SC metabolism being crucial for the instruction of quiescence, proliferation and differentiation processes. OCR can be assessed on isolated mitochondria, tissue homogenate, permeabilized tissue, living cells and tissues (Kelly et al., 2013). Current available methods measure oxygen concentration of the sample over time (oxygraphy). Firstly invented by Clark in 1953 (Clark et al., 1953), sophisticated highly sensitive Clark type oxygen electrodes are now capable of minimizing oxygen diffusion, allowing the instantaneous on-line recording of OCRs (Gnaiger et al., 1995). Such devices, including Oroboros Oygraph, can simultaneously measure oxygen consumption and fluorometric signals, thus providing a valuable tool for coupling high-resolution respirometry with other mitochondrial functions, including ROS and ATP production, mitochondrial membrane potential and mitochondrial Ca^2+^concentration (Doerrier et al., 2018). Other available methods quantify extracellular OCR. The extracellular flux (XF) analyzer instrument (e.g., Seahorse XF24) measures extracellular oxygen consumption by sampling an extremely small volume (about 2 μL) of medium over time, above a monolayer of cells or tissue slices within a petri dish. XF analyzer allows real-time measurements of oxygen consumption by using solid-state fluorescent oxygen biosensors coupled with highly sensitive photodetectors specific for the excitation and emission of oxygen (532/650 nm; Wu et al., 2007). Sequential addition of drugs impairing mitochondrial complex functions, such as complex V inhibitor (i.e., ATP synthase, oligomycin), oxidative phosphorylation uncoupler (FCCP) and complex III and I inhibitors (antimycin A and rotenone), provides information on: (i) the amount of oxygen consumed for ATP production; (ii) basal and the maximal respiration rates; (iii) spare respiratory capacity; (iv) the fraction of oxygen not used for ATP production (proton leak); and (v) the non-mitochondrial oxygen consumption (Salabei et al., 2014). The main limitation of these methods is that they cannot provide any information on the specific metabolic flux (i.e., glucose, glutamine or FAO) contributing to the overall oxygen consumption. Recently, these methods have been proposed for assessing the capability of cultured cells to oxidize specific substrates (i.e., increase oxygen consumption following palmitic acid overload). This measure has been proposed to estimate the metabolic flux for the tested metabolite. However, it only provides a measure of the potential oxidative capability for that metabolite. More precisely, it cannot answer the question as to how much the FAO contributes to a given oxygen consumption. On the contrary, it can give information about how much the tested cell type can maximally oxidize a given substrate. To study the relative contribution of specific metabolic fluxes to the overall oxidative metabolism, the metabolic flux analysis is required.

To study real-time metabolism at a single cell level in living samples, fluorescent metabolic biosensors that are able to bind functional state to a fluorescence protein activity have been developed (Mongeon et al., 2016). Genetically encoded fluorescence and fluorescence resonance energy transfer (FRET) reporters allow real-time measurement of the selective metabolite concentrations and fluxes in intact cells both *in vitro* and *in vivo*. The genetically encoded biosensor Peredox specific to the NADH:NAD+ couple, allows to visualize the cytosolic NADH:NAD+ redox state in cells or tissues using two-photon fluorescence lifetime imaging microscopy (Mongeon et al., 2016). Cytosolic NAD+ is converted to NADH during glycolysis by the glyceraldehyde-3-phosphate dehydrogenase. Moreover, NADH:NAD+ ratio responds to lactate dehydrogenase activity in the cytosol and to mitochondrial NADH shuttles. Interestingly, the fluorescence lifetime of this sensor protein is very short (nanoseconds) giving a direct report of cytosolic NADH:NAD+ ratio (Hung et al., 2011). This probe has been used successfully to measure the increase of neuronal glycolysis triggered by neuronal electrical stimulation (Díaz-García et al., 2017). Similarly, the genetically encoded FRET lactate sensor Laconic allows for monitoring of cellular or tissue lactate dynamics (Machler et al., 2016). The transduction of such probes, driven by the tissue-specific promoters, allows the detection of cell metabolism in a defined subset of cells. Laconic probe was used to study* in vivo* lactate metabolism in astrocytes (Mulder et al., 2016). Laconic was transduced in the primary somatosensory cortex of mice using a viral vector driven by GFAP promoter and therefore expressed in the cytoplasm of protoplasmic cortical astrocytes (Machler et al., 2016). The genetically encoded biosensor Pyronic is a FRET sensor capable of quantitatively measuring pyruvate transport, pyruvate production and mitochondrial pyruvate consumption in living single cell at high temporal resolution. Pyronic has been used in different cell types *in vitro* and *ex vivo* (i.e., HEK293 cells, neurons and astrocytes; San Martín et al., 2014; Lerchundi et al., 2015; Baeza-Lehnert et al., 2019). Central for the understanding of stem and differentiated cell metabolism is the real-time estimation of ATP:ADP ratio. The genetically-encoded fluorescent biosensor, PercevalHR, senses the range of intracellular ATP:ADP (Berg et al., 2009), and it can be used with one- or two-photon microscopy in live samples (Tantama et al., 2013). Similarly to PercevalHR, the ATeam FRET-based biosensors allow for visualizing ATP levels inside a single living cell (Imamura et al., 2009). Both Perceval and ATeam biosensors are sensitive reporters of physiological changes in energy consumption and production and can provide an accurate and real-time estimation of ATP variations when cells are exposed to multiple stimuli (Tantama et al., 2013; Trevisiol et al., 2017). Transgenic mice genetically engineered to produce fluorescent metabolic biosensors in all cells or in specific cell types are now available (Trevisiol et al., 2017). These reporter mice are of great interest to study real-time metabolism at single cell level in living animal (Trevisiol et al., 2017; Baeza-Lehnert et al., 2019).

Biosensors are of great interest for the understanding of cellular metabolism. The major limitation of this novel technique, however, is that it needs gene transfer of the probe, being potentially utilized only in preclinical animal models. Moreover, such *in vivo* studies require accessibility of the tissue for two-photon imaging.

## Conclusion and Perspective

Despite the plethora of methods and protocols that have been published in the last decade, only a limited selection of them are compatible with the constraints imposed by SCs and are therefore suitable for accurate SC metabolic analysis. Both targeted and untargeted approaches provide valuable tools to describe and analyze the cellular metabolism of a specific stem cell population. Given the fast dynamic of intracellular metabolism, the available techniques for sorting (i.e., FACS or immunomagnetic) homogeneous cell population, from a tissue or from a cell suspension, require too long procedures. Therefore, the major limitation is to resolve the characterization of cellular metabolism at the single cell level. While single-cell metabolomics approaches allow, avoiding cell isolation and cell lysis, the measure of SC metabolism, most of the current methodologies, including MS and RMN, require cell separation. Therefore, they provide relevant information on SC metabolism mostly *in vitro* when homotypic SC population are examined. The identification and quantification of specific SC population *in vivo* may be possible using MRS if peculiar metabolites are uniquely enriched in such distinct SC type (Manganas et al., 2007). Furthermore, the development of new biosensors will allow the real-time detection of several metabolites and metabolic fluxes at a single cell level in living samples. The assessment of the modification of the cellular metabolism with time within a certain cell population will also provide important and potentially exploitable information. It would be of great advantage to follow intracellular metabolic adaptation in real time when a defined experimental condition is applied. A further level of analysis will be the assessment of a specific metabolic signature or flux in a subset of cellular compartments, such as lamellipodia (De Bock et al., 2013), lysosome (Abu-Remaileh et al., 2017; Wolfson et al., 2017), mitochondria (Yoshida et al., 2017) or cytoplasm (Pan et al., 2018).

The combination of the different methodologies available nowadays provides wide equipment for the study of cellular metabolism of quiescent, proliferating and differentiating stem cells. Generation of new knowledge on cellular metabolism will hopefully generate novel therapeutic strategies capable of driving specific and desired cellular phenotype.

## Author Contributions

All authors listed have made a substantial, direct and intellectual contribution to the work, and approved it for publication.

## Conflict of Interest Statement

The authors declare that the research was conducted in the absence of any commercial or financial relationships that could be construed as a potential conflict of interest.
